# Effect of intermediate-term firewood smoke air pollution on cardiometabolic risk factors and inflammatory markers

**DOI:** 10.3389/fcvm.2023.1252542

**Published:** 2023-11-21

**Authors:** Fernando Lanas, Nicolás Saavedra, Kathleen Saavedra, Montserrat Hevia, Pamela Seron, Luis A. Salazar

**Affiliations:** ^1^ Department of Internal Medicine, Universidad de La Frontera, Temuco, Chile; ^2^ Department of Basic Sciences, Universidad de La Frontera, Temuco, Chile

**Keywords:** air pollution, cardiometabolic risk factors, inflammatory cytokines, cold weather, cardiovascular risk

## Abstract

**Background:**

Temuco is a city in Southern Chile with elevated levels of air pollution (AP), mainly due to using wood as combustion throughout the cold season. The study aimed to assess the differences in cardiometabolic risk factors, estimated cardiovascular risk, and blood level of inflammatory markers between high AP (HAP) and low AP (LAP) periods.

**Methods:**

A prospective panel study was conducted between January to September 2018. Air pollution was assessed by PM_2.5_ concentration. Ninety individuals from the general population were included in the study. Measurements were performed in the HAP and LAP, including medical history and lifestyle, physical activity assessment, physical exam, and fasting blood samples for glucose, lipids, and circulatory inflammatory mediators.

**Results:**

In the high air pollution period, systolic blood pressure was 3 mmHg higher (*p* = 0.05). HDL-cholesterol was 14.2 mg/dl lower (*p* < 0.001), Framingham risk score increased from 14.5 to 18.0 (*p* < 0.001), and highly significant lower levels of interleukins, MCP1, MMP1, MMP2, sICAM, and svCAM were observed.

**Conclusions:**

HAP was associated with increased cardiometabolic risk factors and estimated cardiovascular risk. However, a lower level of circulating acute inflammatory molecules was observed. Inflammatory molecules blood levels were not associated with changes in cardiometabolic risk factors.

## 1. Introduction

The Global Burden of Disease (GBD) study estimates that 4.2 million deaths were attributable to fine particulate material (PM_2.5_) air pollution in 2015, most of them in low- and middle-income countries, where the exposure has grown significantly (1). In 2019, 70,668 deaths and 1,736,414 DALYs due to CVD were attributed to total PM_2.5_ exposure in South America (2). According to GBD estimates 21% of deaths from cardiovascular disease and 24% of stroke deaths are attributable to air pollution (1). WHO has reported that 36% of deaths attributable to air pollution are due to ischemic heart disease and another 36% to stroke (3). Both acute and long-term exposure to air pollution has been associated with cardiovascular events. A systematic review reported that short-term PM_2.5_ exposure increased the relative risk for acute myocardial infarction by 2.5% per 10 µg/m^3^ increase (4), and another study estimated a long-term pooled effect of an 11% increase in cardiovascular mortality for each 10 µg/m^3^ PM_2.5_ increase (5). The main proposed pathways linking air pollution to cardiovascular diseases are the induction of oxidative stress leading to systemic inflammation and atherosclerosis (6).

Several studies in animal models and *in vitro* have also reported that the adverse effects of PM on the cardiovascular system are by eliciting oxidative stress and inflammation (7). Oxidative stress induces endothelial function disorders, smooth muscle cell proliferation, macrophage recruitment, and inflammation, all essential factors in atherosclerotic plaque formation (8). For example, short PM exposure in elderly subjects with coronary artery disease produces elevated levels of inflammatory molecules (TNF-, Il-6, ICAM-1, VCAM-1, and P- selectin), platelet activation, and reduced levels of antioxidant enzymes Superoxide Dismutase and Glutathione Peroxidase (9). Another study in 93 elderly non-smoking adults suggested that short-term exposures to PM_2.5_ pollutants (black carbon, NOx, and CO) with a high oxidative potential contribute to microvascular endothelial dysfunction (10). However, recent reports have demonstrated no clear systemic inflammatory response on controlled exposure to PM_2.5_ and PM_10_, concentrated ambient particles, or dilute diesel exhaust (11, 12).

Association between air pollution and the progression of atherosclerosis assessed through carotid intima-media thickness (13) and coronary calcium (14) have been reported. The potential mechanism for accelerated atherosclerosis may involve endothelial injury and dysfunction (15) and the worsening of cardiometabolic risk factors. Furthermore, short and long-term exposure to air pollution is associated with increased blood pressure (16–18) and elevated fasting glucose levels (19). Changes in the blood lipids profile have also been reported with long-term air pollution exposure (20, 21).

**Figure 1:**
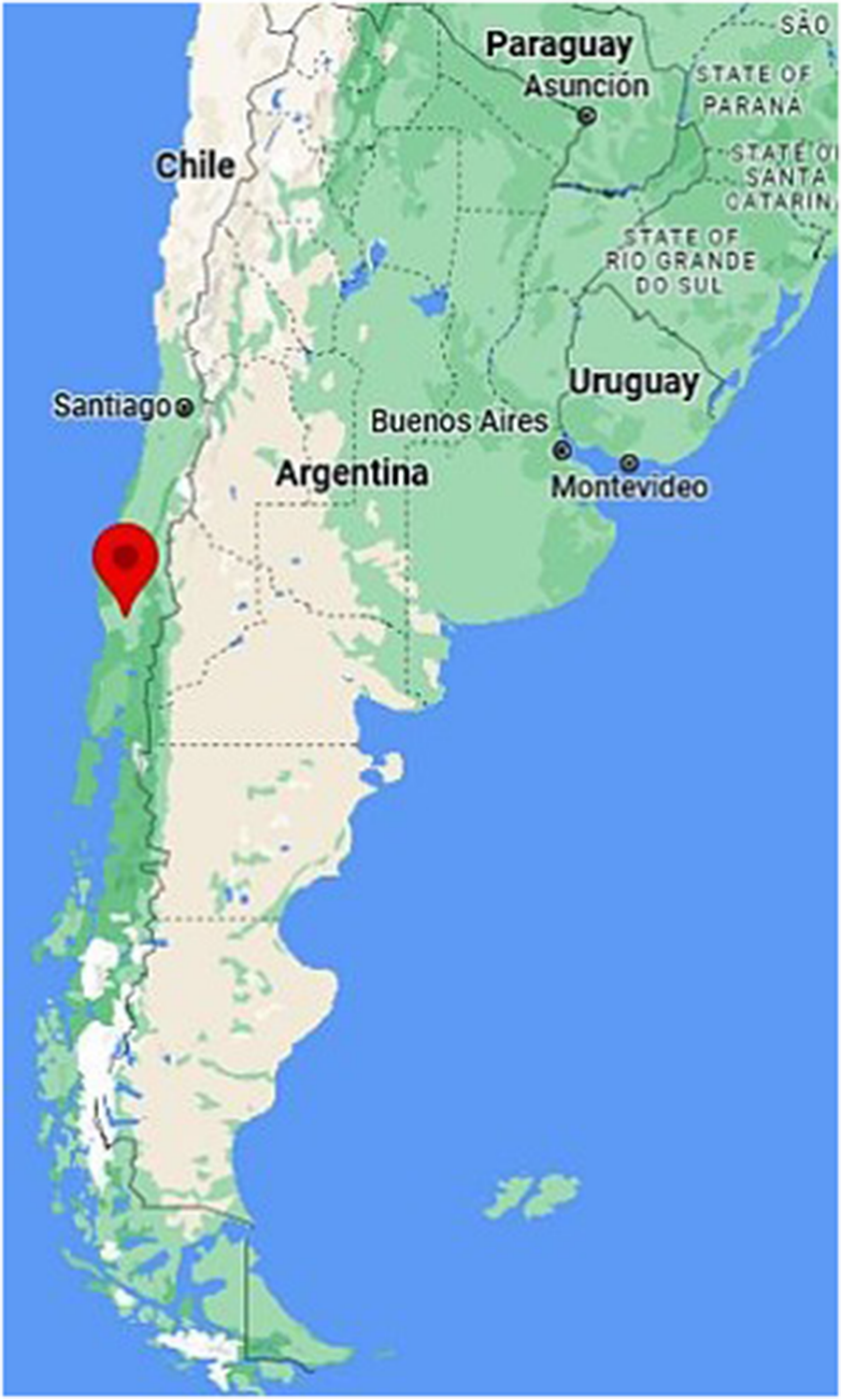
Geographical location of Temuco city, Chile.

A significant contributor to air pollution in developing countries is biomass burning. This study was conducted in Temuco, a city in Southern Chile (Figure 1) with one of the highest levels of air pollution among American cities, mainly due to using wood as the principal combustion source throughout the cold season (April to September). PM_10_ levels were significantly associated with daily mortality and morbidity in Temuco, with older people presenting a higher risk (22). In the PURE cohort, where Temuco is one of the participant sites, the population attributable fraction for PM_2·5_ was 13·9% for cardiovascular disease events, 8·4% for myocardial infarction, 19·6% for stroke, and 8·3% for cardiovascular disease mortality (23). This study aimed to assess the differences in cardiometabolic risk factors, estimated cardiovascular risk, and blood level of inflammatory markers between high air pollution (HAP) and low air pollution (LAP) periods.

## 2. Materials and methods

### 2.1. Study design and participants

A prospective panel study was conducted between January and September 2018 in Temuco, Chile, including ninety healthy individuals older than 35 years without cardiovascular disease or recent acute infection, randomly selected from the 2,253 urban participants in the Prospective Urban and Rural Epidemiology (PURE) cohort. The Temuco-PURE study is part of an international cohort (24), selected using a random sampling stratified by socioeconomic status, assembled in urban Temuco between 2006 and 2008, that has been followed annually. The sample size was calculated assuming we will observe at least half of the increase in IL-6 reported in individuals exposed to air pollution (25). With alpha 0.05, power of 90%, a difference in IL-6 of 9.3 μm/ml, and a standard deviation of 11.5 μm/ml, a total sample size of 82 was obtained, and a 10% was added for eventual losses of follow-up. All included participants were asked to sign an informed consent, which was previously approved by Universidad de La Frontera Ethics Committee (N°042_17). Clinical and biochemical data were obtained at the end of each subject's LAP and HAP. LAP measurements were performed between January and March, while HAP measurements were performed at the end of the cold season from August to September when air pollution is significantly higher due to using firewood as combustion. Air pollution levels at LAP and HAP were estimated using publicly available data from three monitoring stations operated by the National Air Quality Information System (SINCA) of Ministerio del Medio Ambiente, Gobierno de Chile. Monthly mean concentrations of PM_2.5_ and PM_10_ from these three monitoring stations were used to represent residents' air pollution exposure. A PM_2.5_ of 10 µg/m^3^ was considered a limit between LAP and HAP following WHO global air quality guidelines (26).

### 2.2. Clinical, demographic, and laboratory measurements

Clinical measurements included questionnaires about demographics, lifestyle (smoking and physical activity), health, and medication use. Physical activity was assessed with a short version of the International Physical Activity Questionnaire (IPAQ) validated in Spanish (27). Measurements of weight, height, blood pressure, heart rate, hip and waist perimeter were obtained. Blood pressure was assessed using automatic devices following international recommendations, and the average of two measurements was recorded. The room temperature was maintained at 20°C to avoid the influence of temperature on blood pressure.

Fasting venous peripheral blood samples were drawn at the end of the LAP and HAP to obtain serum samples. Serum glucose, triglycerides, total cholesterol, HDL cholesterol, LDL cholesterol, and creatinine were measured using colorimetric methods. Cardiovascular risk was estimated at each period using the Framingham equation, including age, gender, systolic blood pressure, smoking, total and HDL cholesterol, hypertension treatment, and diabetes (28). Circulating inflammatory mediators, including Interleukin (IL)-6, IL-10, IL-18, Matrix metalloproteinase (MMP)1, MMP-2, Monocyte chemoattractant protein 1 (MCP-1), soluble vascular cell adhesion molecule (sVCAM), and serum levels of soluble intercellular adhesion molecule (sICAM) were quantified using a MAGPIX® System using MILLIPLEX® MAP magnetic bead-based multi-analyte panels.

### 2.3. Statistical analyses

Summaries by group using appropriate descriptive statistics are provided for study variables, including demographic, clinical, and laboratory measurements. Descriptive statistics such as mean, median, standard deviation, minimum, and maximum are used to summarize continuous variables. Counts and percentages are used to summarize categorical variables. Mean values were compared between LAP and HAP periods with the paired *t*-test for continuous values. Proportions were compared by chi-square (or Fisher's exact). The effect of the medication on outcomes was assessed with stratified analyses. Correlations between clinical and laboratory variables and inflammatory markers were evaluated by the Spearman method. Statistical analyses was performed with STATA 16,1® (Statacorp, Tx. EE. UU.).

## 3. Results

### 3.1. Air pollution at high and low air pollution periods

**Figure 2:**
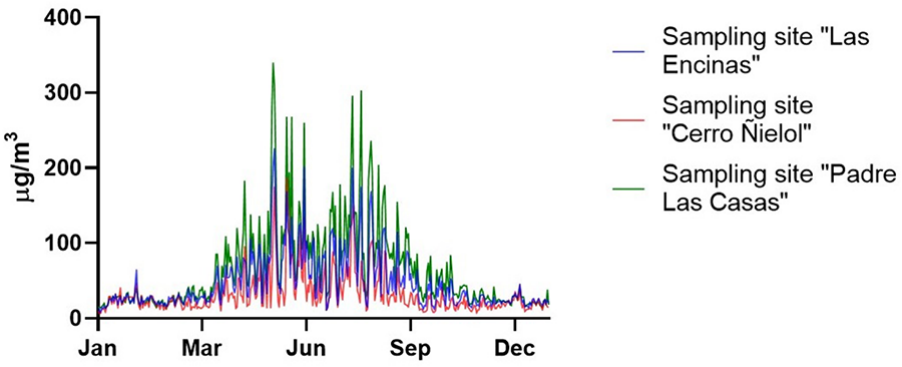
Daily PM 2.5 Concentrations in Temuco city during 2018.

Air pollution in 2018 assessed by PM_2.5_ concentration was low between January and March, higher between April and September (the cold-weather season), and PM_2.5_ concentration returned to low values after September (Figure 2). PM_2.5_ ranges from 2.97 to 8.98 (μg/m^3^) in the period of LAP sampling and between 50.39 and 63.79 (μg/m^3^) in the months of HAP sampling (Table 1, Figure 2).

**Table 1:** Monthly average of PM_2.5_, PM_10_, temperature and humidity in Temuco, 2018.

Parameter	Jan	Feb	Mar	Jul	Aug
PM 2.5 (µg/m^3^)	3.61	2.97	8.98	63.79	50.39
PM 10 (µg/m^3^)	23.1	20.8	21.92	71.23	57.88
Temperature (°C)	16.7	17.3	14.1	7.1	7.9
Relative humidity (%)	72.24	75	82	89	89

PM, particulate material.

### 3.2. Clinical, demographic and laboratory measurements

Ninety individuals were included in the study; the mean age was 52 ± 10.1 years old, ranging from 36 to 75 years old. Forty-four were males, and 46 were females. Thirty-five had hypertension in treatment, 9 had diabetes mellitus, eight used statins, and 20 were active smokers.

Systolic blood pressure was 3 mmHg higher in the HAP period in the overall sample; in those without blood pressure medication, blood pressure was 117.1 ± 11.2 mmHg in the LAP period and 121.5 ± 13.3 mmHg in the HAP period (*p* = 0.0024) (Table 2). Total cholesterol was significantly higher in the LAP period due to higher HDL cholesterol values. In those without statin use, total cholesterol was 206.4 ± 36.0 mg/dl and 195.3 ± 43.1 mg/dl (*p* = 0.0041), and HDL cholesterol was 58.7 ± 14.6 mg/dl and 44.3 ± 13.7 mg/dl (*p* ≤ 0.0001) in LAP and HAP periods respectively. Due to the difference in blood pressure and HDL cholesterol levels, the estimated 10-year risk of cardiovascular events was higher in the HAP period. No significant difference between the LAP and HAP periods was observed in diastolic blood pressure, LDL cholesterol, or glucose levels in the overall sample and those without specific medication (Table 2). Highly significant lower levels of interleukins, MCP1, MMP1, MMP2, sICAM, and svCAM were observed during the HAP period (Table 3). Blood pressure, glucose medication, and statin use did not modify the results.

**Table 2:** Mean value and standard deviation of clinical and laboratory values and 10-years cardiovascular risk estimation.

	Low AP period	High AP period	% Change	*p* value
Systolic BP (mmHg)	126.9 ± 20.1	129.8 ± 20.5	2.2	0.050
Diastolic BP (mmHg)	81.0 ± 11.6	79.8 ± 12.2	−1.5	0.31
Weight (Kg)	78.3 ± 14.6	77.9 ± 14.0	−0.5	0.24
BMI (Kg/m^2^)	29.3 ± 4.6	29.4 ± 4.4	0	0.18
Waist (cm)	96.2 ± 11.6	96.0 ± 10.8	−0.2	0.50
WTH ratio	0.94 ± 0.07	0.92 ± 0.07	−0.2	0.13
Total cholesterol (mg/dl)	206.3 ± 38.9	193.9 ± 43.6	−0.06	0.0023
HDL-cholesterol (mg/dl)	58.8 ± 14.2	44.6 ± 13.4	31.8	<0.001
LDL-cholesterol	128.6 ± 30.4	123.1 ± 34.9	4.4	0.13
Triglycerides (mg/dl)	169.1 ± 113.1	140.4 ± 85.6	−20.4	0.0006
Glucose (mg/dl)	104.0 ± 35.3	102.5 ± 34.6	−1.4	0.41
METs/week	2,701.9 ± 3,336.8	3,206.9 ± 4,141.5	15.7	0.19
Framingham score	14.5 ± 13.2	18.0 ± 15.5	19.4	<0.0001

Data are reported as mean ± standard deviation, AP, air pollution; BP, blood pressure; BMI, body mass index; WTH, waist to hip ratio; HDL, high density lipoprotein; LDL, low density lipoprotein; METs, metabolic equivalents.

**Table 3:** Mean value and standard deviation of inflammatory markers in the low and high air pollution periods.

Parameter	Low AP period	High AP period	% Change	*p* value
IL 6 (pg/ml)	18.0 ± 6.4	13.3 ± 4.8	−35.3	<0.0001
IL 10 (pg/ml)	3.6 ± 0.9	2.9 ± 1.0	−24.1	<0.001
IL 18 (pg/ml)	66.3 ± 18.0	38.5 ± 18.0	−72.2	<0.0001
MCP1 (pg/ml)	178.2 ± 114.6	67.8 ± 58.7	−152.8	<0.0001
MMP1 (pg/ml)	263.9 ± 316.4	107.5 ± 145.6	−145.4	<0.0001
MMP2 (pg/ml)	945.9 ± 394.7	344.7 ± 245.0	−174.7	<0.0001
sICAM (pg/ml)	2,70,502.8 ± 1,97,273.7	1,51,436.7 ± 1,17,310.6	−78.6	<0.0001
svCAM (pg/ml)	20,011.0 ± 10,480.6	12,762.9 ± 4,934.8	−56.8	<0.0001

Data are reported as mean ± standard deviation. AP, air pollution; IL, interleukin; MCP1, monocyte chemoattractant protein 1; MMP1, matrix metalloproteinase-1; MMP2, matrix metalloproteinase-2; sICAM, serum levels of soluble intercellular adhesion molecule; svCAM, soluble vascular cell adhesion molecule.

There was a moderate to high correlation between the changes observed in cytokines and a moderate correlation between cytokines changes and MMP2, sICAM, and svCAM changes. Also, changes in sICAM and svCAM were moderately correlated (Table 4). No significant correlation was observed in changes in systolic blood pressure and total and HDL cholesterol with the variations observed in inflammatory markers.

**Table 4:** Correlation matrix between the changes observed in cytokines levels (pg/ml) spearman's rank correlation coefficient.

Parameter	IL 6	IL 10	IL 18	MCP1	MMP1	MMP2	sICAM	svCAM
IL 6	1							
IL 10	0.75*	1						
IL 18	0.60*	0.50*	1					
MCP1	0.31*	0.31*	0.35*	1				
MMP1	0.12	0.09	0.27*	0.42*	1			
MMP2	0.58*	0.60*	0.49*	0.25*	0.10	1		
sICAM	0.60*	0.46*	0.41*	0.17	0.15	0.51*	1	
svCAM	0.49*	0.52*	0.47*	0.35*	0.19	0.62*	0.49	1

IL, interleukin; MCP1, monocyte chemoattractant protein 1; MMP1, matrix metalloproteinase-1; MMP2, matrix metalloproteinase-2; sICAM, serum levels of soluble intercellular adhesion molecule; svCAM, soluble vascular cell adhesion molecule.

* *p* < 0.05.

## 4. Discussion

Our main results are that after an intermediate exposure time, 3–4 months, of mainly wood smoke air pollution, reaching PM_2.5_ mean monthly values between 50,39 to 63,79 μg/m^3^, there is an increase in systolic blood pressure, a decrease in HDL cholesterol, and an increased estimated 10 years cardiovascular risk. Also, all inflammatory markers were significantly lower during the HAP period, but these changes were not correlated with the changes in cardiometabolic risk factors or estimated cardiovascular risk.

Residential wood combustion is a significant source of particulate air pollution in many countries, and biomass combustion emissions are expected to increase in the following years. In contrast, emissions from motor vehicles will decline due to improved technologies and stringent regulations, and information regarding wood smoke toxicity is limited compared to fossil fuel combustion (29). Wood smoke particles' chemical composition is different from those derived from fossil fuel combustion. Additionally, wood smoke exposures are highly variable due to the multifactorial nature of wood smoke creation (30). A comparative effect of diesel and gasoline engine exhausts, hardwood smoke, and simulated coal emissions in experimental animals concluded that all exposures caused significant results and that each could be deemed most or least toxic depending on the exposure metric used for comparison (31). There is evidence that wood smoke particles can induce an inflammatory response (32, 33) and cardiovascular events (34).

The main change in cardiometabolic risk factors in our results was the HDL-C decrease during the high pollution period, from 58 to 44 mg/dl, with an increased LDL/HDL cholesterol ratio. The Study of Women's Health Across the Nation has reported a similar effect of PM_2.5_ increase, a −0.7% in high-density lipoprotein cholesterols for each 3 μg/m^3^ increase of 1-year PM_2.5_ exposure (21). Also, similarly to our results, a decrease in HDL cholesterol and triglyceride levels has been reported in China (20) and in an analyses of the UK Biobank (35).

We observed an increase in blood pressure of 2.9 mmHg in systolic blood pressure and a mean difference in the PM_2.5_ between LAP and HAP periods of 52 μg/m^3^, without a difference in diastolic blood pressure. PM causes a systemic inflammatory response and autonomic dysfunction, which may lead to elevated blood pressure (36). A meta-analysis reported that increases in ambient PM_2.5_ by 10 μg/m^3^ are associated with 1–3 mmHg elevations in systolic blood pressure (16), a higher magnitude than the one we observed in our participants. However, in another meta-analysis that assessed the combined estimate of studies with a panel design, excluding cross-sectional studies, the reported increase in systolic blood pressure was lower, 0.961 mmHg (95% CI 0.497–1.426) (17), a study performed in Barcelona reported a rise of 1.37 mmHg 24-h DBP and 1.48 mmHg daytime DBP for each increase of 10 μg/m^3^ of PM_10_ (37) and other studies did not find an association (38). While it has been reported an effect of temperature on blood pressure in our study, indoor temperature was set to 20°C throughout the year, reducing changes due to lower winter temperatures (39).

We did not observe differences in blood glucose levels between the LAP and HAP periods. Studies have found that ambient particulate affects fasting blood glucose. However, the results are not consistent. In a recent meta-analysis, fasting blood glucose increased 0.23 mmol/L per 10 μg/m^3^ of increased PM_2.5_ after long-term exposure and 0.08 mmol/L after short-term exposure (19), but in a meta-analysis restricted to cohort studies, a more appropriate study design, no association was observed between PM_2.5_ levels and insulin resistance or glucose levels (40). Our results observed an increase in estimated cardiovascular risk Framingham score of 19.4%, related to the rise in systolic BP and decreased HDL cholesterol. A recent study reported an increase in daily cardiovascular mortality of 0.55% with each increase of 10 μg/m^3^ in the moving average PM_2.5_ in 652 cities (41). However, in our analyses, no significant correlation was observed between changes in systolic blood pressure and total and HDL cholesterol blood levels with the variations observed in inflammatory markers. This result suggests that the observed changes in blood pressure and HDL cholesterol may not be due to differences in air pollution but may be associated with changes in physical activity, diet, or lower temperatures during the HAP period.

In our study, we observed an inverse effect of PM_2.5_ in circulating inflammatory mediators: IL-6, IL-10, IL-18, MMP-1, MMP-2, MCP-1, sVCAM, and sICAM levels decreased during HAP, while the production of these markers of inflammation increased during LAP. A recent meta-analysis assessed short-and long-term associations of particulate matter with inflammation markers in 44 studies. They reported significant changes in TNF-α and fibrinogen with short-term PM_2.5_ exposure and no significant differences in IL-6 and IL-8 with short-term exposure. A reduction of IL-6 and IL-8 was observed in 4 of 11 and 2 of 7 studies, respectively. Long-term analyses were not possible due to limited information. A marked geographical effect was observed: IL-6 was significantly associated with PM25 exposure in Asia but not in Europe. An additional meta-regression analysis to assess the causes of results heterogeneity showed that air pollutant levels, age, study location, disease status, and study design might be the source (25).

Additionally, we must consider that our study participants were exposed to PM and other not-measured gaseous air pollutants that may influence inflammatory response. A 2022 meta-analysis analyzed the association between ozone, nitrogen dioxide, sulfur dioxide, carbon dioxide, and major inflammatory biomarkers, including IL-6 and TNF-α. They concluded that there were significant positive associations between short-term but not long-term exposure to gaseous air pollutants and inflammatory biomarkers, and several studies reported IL-6 levels reduction with air pollution (42). The differences between studies- including ours- may be explained by different PM components between countries, time, concomitant gaseous components and levels of exposure, participants' characteristics, and study design.

The strengths of our study are the sample's representativeness, since participants were a random sample of the city population, and the standardized participant measurements implemented in the PURE study. Also, the study design, where the same individual is his control, avoids potential bias. The limitations are related to the ecologic design, where exposure was not measured at the individual level and did not include indoor air pollution assessment. However, a recent study in the same city reported similar median PM_2.5_ between indoor and outdoor concentrations: 44.4 and 41.8, respectively (43). Also, we did not include other air contaminant molecules described in wood smoke pollution because the information was unavailable. However, several studies have reported that they correlate well with PM_2.5_ concentration (44). Our cohort study age limits, between 36 and 75 years old, exclude younger or older age individuals who may exhibit different blood pressure, cholesterol, glucose levels, or changes in cytokine levels. However, they represent an age range group where most cardiovascular events occur. Future studies must include younger individuals. Additionally, given that air pollution exposure was limited to PM2.5 and the panel design of the study, our results needs to be considered preliminary.

## 5. Conclusions

HAP was associated with increased systolic blood pressure, and estimated Framingham cardiovascular and decreased HDL cholesterol and inflammatory markers. The absence of correlation between the changes observed in traditional cardiovascular risk factors and cytokine levels suggests additional causal factors related to lifestyle during the cold season. Under this hypothesis, the increased event rate of cardiovascular events observed with air pollution can be the consequence of both the inflammatory effect of air pollution and the lifestyle changes that modify traditional risk factors. Health policies to control air pollution and improve traditional risk factor control should be implemented with the aim of reducing the significant increase in cardiovascular events observed during the cold season.

## Data Availability

The original contributions presented in the study are included in the article/Supplementary Material, further inquiries can be directed to the corresponding author.
